# Single-cell and spatial atlas of glioblastoma heterogeneity: characterizing the *PCLAF*+ subtype and *YEATS4*’s oncogenic role

**DOI:** 10.3389/fimmu.2025.1614549

**Published:** 2025-07-25

**Authors:** Siqi Ma, Yuwei Sun, Shaowei Zheng, Yilong Fu, Liangyu Wang, Dun Liu, Henan Jiao, Xuqiang Zhu, Xueyuan Li, Dongming Yan, Di Chen, Zi Ye

**Affiliations:** ^1^ Department of Neurosurgery, The First Affiliated Hospital of Zhengzhou University, Zhengzhou University, Zhengzhou, Henan, China; ^2^ College of First Clinical Medicine, Shandong University of Traditional Chinese Medicine, Jinan, Shandong, China; ^3^ Department of Scientific Research, The First Affiliated Hospital of Zhengzhou University, Zhengzhou University, Zhengzhou, Henan, China

**Keywords:** glioblastoma, scRNA-seq, spatial transcriptomics, YEATS4, tumor heterogeneity

## Abstract

**Background:**

Glioblastoma (GBM) was considered the most aggressive type of primary brain tumor, marked by poor clinical outcomes and a high tendency to relapse. The therapeutic efficacy of GBM was significantly compromised by tumor heterogeneity, dysregulated metabolic pathways, the formation of an immunosuppressive microenvironment, and treatment resistance. Therefore, multi-dimensional therapeutic strategies targeting GBM-specific molecular features, its intrinsic properties, and microenvironmental regulatory networks were considered to potentially provide new breakthroughs for overcoming treatment resistance in GBM.

**Methods:**

We analyzed single-cell RNA sequencing (scRNA-seq) data processed with the Seurat package to accurately identify cell types. Spatial transcriptomics integrated Multimodal Intersection Analysis, TransferData, and Robust Cell Type Decomposition techniques to characterize the spatial distribution patterns of key cell subtypes. CellChat was employed to assess intercellular communication networks. Furthermore, *in vitro* experiments confirmed the main regulatory role of YEATS4 (key transcription factor of C2 *PCLAF*+ subtype) in GBM malignant progression.

**Results:**

Through scRNA-seq, we identified the C2 *PCLAF*+ subtype in GBM and analyzed its molecular characteristics and functional role in tumor progression. This subtype exhibited a unique malignant phenotype, marked by significant proliferative activity, characteristic metabolic reprogramming, and dysregulated cell death regulation mechanisms. Spatial transcriptomics revealed its preferential localization within specific tumor niches. Furthermore, the C2 *PCLAF*+ subtype established a specific interaction with fibroblasts through the MDK-LRP1 ligand–receptor pair. Critically, silencing *YEATS4 in vitro* significantly inhibited GBM malignancy. Additionally, the prognostic risk score model based on the C2 *PCLAF*+ subtype demonstrated significant clinical translational value.

**Conclusion:**

Our study systematically elucidated the malignant characteristics of the C2 *PCLAF*+ subtype and its molecular mechanisms driving GBM progression. This subtype promoted therapeutic resistance through unique metabolic reprogramming, MDK-LRP1-mediated microenvironmental interactions, and immunosuppressive properties. *YEATS4* knockdown effectively suppressed malignant tumor behaviors, highlighting its therapeutic potential. These findings provided novel targeted intervention strategies to address GBM heterogeneity and treatment resistance, offering promising avenues for overcoming current therapeutic limitations.

## Introduction

Glioblastoma (GBM) was recognized as the most frequently diagnosed and aggressive primary brain malignancy (1), responsible for more than 48% of cases (2) and occurring at a rate of approximately 3.2 per 100,000 people each year (3). GBM predominantly affected individuals aged 50 years and older (4), with incidence figures being notably elevated in male patients (2). GBM was associated with high mortality and poor prognosis, exhibiting a 5-year survival rate of approximately 5% (5) and a median survival of only about 15 months (6, 7). Based on clinical status and disease progression, GBM was categorized into two types: newly diagnosed Glioblastoma (ndGBM) and recurrent Glioblastoma (rGBM) (8). NdGBM exhibited highly malignant and rapidly proliferative biological characteristics (1), while rGBM not only demonstrated reduced sensitivity to chemoradiotherapy but also typically displayed more advanced malignancy (9, 10).

For GBM patients, the Stupp treatment paradigm typically comprised initial surgical intervention, followed by concurrent radiotherapy and chemotherapy, and subsequent adjuvant chemotherapy (1, 11). However, it was associated with a high recurrence rate, treatment resistance, and poor prognosis (12, 13). In recent years, emerging strategies such as immunotherapy (e.g., PD-1 inhibitors) (14), targeted metabolic pathways (e.g., IDH mutations) (15), and nanoparticle drug delivery systems (16, 17) had made some progress. However, these approaches were still limited by the blood-brain barrier (BBB), immunosuppressive tumor microenvironment (TME), and resistance (12, 18, 19). Additionally, the incidence of GBM had significantly increased in elderly patients, and their prognosis was worse, further highlighting the challenges in treating GBM. Therefore, it was of great significance to explore the molecular mechanisms of GBM, resistance, and the regulation of the microenvironment to develop novel therapeutic strategies.

The breakthrough advancement of single-cell RNA sequencing (scRNA-seq) technology granted unprecedented high-definition analytical capabilities to GBM research (20–22). This technology enabled the precise identification of key cell subtypes and immune microenvironment characteristics, offering important insights into the molecular mechanisms underlying tumor recurrence and resistance. Meanwhile, scRNA-seq could dynamically monitor the evolution of tumor cell heterogeneity and microenvironment remodeling during treatment, providing molecular clues for adjusting personalized treatment strategies. The introduction of spatial transcriptomics (ST) further expanded the analytical dimensions, through its unique capacity to resolve the spatial architecture of diverse cell types in GBM tissues, especially the spatial enrichment patterns of key cell subtypes in specific tumor niches.

This study systematically analyzed the cellular heterogeneity characteristics of GBM through integrated scRNA-seq and ST technologies. We identified the C2 *PCLAF*+ as a critical driver of tumor progression, characterized by pronounced proliferative activity, metabolic reprogramming (such as enhanced oxidative phosphorylation and glycolysis), and distinct cell death regulation, collectively contributed to its resistance to treatment. ST analysis further showed that this subtype had distinct distribution patterns in specific tumor niches, significantly co-localizing with regions of high heterogeneity and proliferative activity. We also elucidated the interaction mechanism between the C2 *PCLAF*+ subtype and fibroblasts through the MDK-LRP1 ligand–receptor pair and identified key transcription factors (TFs), such as YEATS4, that regulated its malignant phenotype. Functional validation demonstrated that silencing *YEATS4* significantly inhibited GBM cell proliferation, migration, and invasion. Guided by these findings, we formulated a prognostic risk scoring model that effectively distinguished patient outcomes and correlated with specific immune microenvironment characteristics, genomic variation patterns, and drug sensitivity. Our findings not only deepened the understanding of the mechanisms underlying GBM heterogeneity but also provided important theoretical foundations and potential targets for developing precision therapies targeting specific subtypes.

## Materials and methods

### Collection of GBM data

We retrieved data from the Gene Expression Omnibus (GEO) database (https://www.ncbi.nlm.nih.gov/geo/) under accession number GSE182109. Following data acquisition, 16 GBM samples were selected for subsequent analysis. To complement our findings, bulk RNA-seq datasets were obtained from The Cancer Genome Atlas (TCGA) website (https://portal.gdc.cancer.gov/). Meanwhile, we conducted an external validation using the dataset with GSE43378. As all data were sourced from publicly available repositories, no additional ethical approval was required for this study.

### Quality control and computational analysis pipeline for scRNA-seq data

First, the initial processing of data was conducted using R (v4.3.3) and Seurat (v4.3.0) (23–25). Then, low-quality cells were eliminated through purification, filtration, and doublet exclusion in accordance with these standards: (1) nFeature detection threshold (300–5,000); (2) nCount detection threshold (500–50,000); (3) mitochondrial gene expression ratio (≤10%); (4) erythrocyte gene expression ratio (≤5%). This stringent quality control process resulted in the retention of 105,873 high-quality cells for subsequent analysis.

We first normalized the raw expression data using the “NormalizeData” function (26–28) to eliminate technical biases such as sequencing depth. Subsequently, we identified the top 2,000 highly variable genes with “FindVariableFeatures” (29, 30) to capture the most biologically significant expression differences between cells (31, 32). Following this, we applied “ScaleData” to standardize all genes, ensuring comparability across different expression levels (33). Next, we performed Principal Component Analysis (PCA) (34, 35) and corrected for batch effects using the Harmony R package (v0.1.1), improving data integration quality (36–38). Based on the top 30 principal components, we further conducted clustering analysis with “FindNeighbors” (39–41) and “FindClusters” (42–44), and finally achieved nonlinear dimensionality reduction and visualization using Uniform Manifold Approximation and Projection (UMAP) (45–47).

### Cell subtype annotation and characterization

Based on the cell clustering results, we systematically annotated and characterized cell subtypes by calculating the mean expression levels of cell-type-specific marker genes obtained from the CellMarker database (http://xteam.xbio.top/CellMarker/).

### Functional enrichment and AUCell analysis

We first systematically identified differentially expressed genes (DEGs) among cell subtypes using “FindAllMarkers” (48, 49), followed by comprehensive functional characterization of these DEGs with the “ClusterProfiler” package. Specifically, we simultaneously analyzed Gene Ontology (GO) terms (50–52) and Kyoto Encyclopedia of Genes and Genomes (KEGG) pathway enrichment (53–56) to elucidate key biological processes and signaling pathways significantly associated with the DEGs. To gain deeper functional insights, we employed Gene Set Enrichment Analysis (GSEA) to evaluate coordinated expression patterns of predefined gene sets (57–59). Furthermore, we implemented the AUCell algorithm to calculate gene activity and enrichment scores for each cell subtype, providing quantitative functional characterization at single-cell resolution.

### Analysis of ST

We selected two samples from the ST dataset (GSE194329). For the ST 1 slide, we identified spatial enrichment patterns of key cell subtypes using Multimodal Intersection Analysis (MIA) and performed spatial localization with Seurat’s TransferData function. The ST 2 slide was analyzed using the Robust Cell Type Decomposition (RCTD) technology to map the key cell subtypes identified in the scRNA-seq data to the ST data, thereby further characterizing their spatial distribution features.

### Differentiation trajectory analysis

We systematically investigated the developmental dynamics of glioma subtypes through multi-dimensional analyses. First, CytoTRACE was employed to evaluate the stemness levels across distinct glioma subtypes. To validate these findings, multilineage trajectory inference was performed with Slingshot (v2.6.0), where developmental paths were determined using the “getLineages” and smooth curves were generated via “getCurves”, ultimately visualizing the trajectories in UMAP. The identification of trajectory initiation and termination points was based on established biological characteristics of distinct glioma subtypes and their associated molecular signatures.

### Intercellular communication network

We systematically deciphered the cell-cell interaction network within the GBM communication microenvironment using CellChat (v1.6.1) to reconstruct intercellular communication networks (60–62). The “netVisual_diffInteraction” was employed to visualize differential communication strength patterns among distinct cell types. Based on the CellChatDB database, we identified significantly activated signaling pathways and specific ligand-receptor regulatory networks.

### Transcriptome regulatory landscape profiling

We employed pySCENIC (v0.10.0) to infer both the clustering of glioma cell subtypes in GBM and their specific transcriptional regulatory modules. Regulatory activity was quantified using AUCell, with significance determined through 100 permutation tests.

### Cell culture

LN229 and A1207 GBM cell lines were propagated in DMEM containing 10% fetal bovine serum (FBS) and 1% penicillin-streptomycin (P/S) at 37°C in a humidified 5% CO_2_ atmosphere.

### Cell transfection

We employed siRNA synthesized by GenePharma (Suzhou, China) to target and silence *YEATS4*. For the transfection experiments, cells were incubated in 6-well plates until reaching 50% confluence, followed by transfection via the Lipofectamine 3000 RNAiMAX system (Invitrogen, USA). Two specific siRNA sequences were designed: si*YEATS4*-1 (GUGUAAGAAUGGAUGCTAU) and si*YEATS4*-2 (AAUCCUUUAAGAGUUGUUA), with non-targeting siRNA (si-NC) serving as the negative control.

### Cell viability monitoring

Cells were seeded in 96-well plates at a density of 5×10³ cells per well and cultured for 24 h. Subsequently, 10 μL of CCK-8 reagent (A311-01, Vazyme) was added to each well, followed by incubation for 2 h at 37°C in the dark. Absorbance was measured at 450 nm using a microplate reader (A33978, Thermo). The experiment was conducted daily for 4 consecutive days, with the results presented as time-viability curves to illustrate dynamic changes.

### Quantitative real-time polymerase chain reaction

RNA was extracted from cells using TRIzol reagent, followed by reverse transcription with the PrimeScript™ Kit. Real-time monitoring was carried out using a fluorescent dye. The primer sequences were as follows: F: TGTTCAAGAGAATGGCCGAA, R: CCACTGATGAGTGTGCCCAT.

### 5-ethynyl-2’-deoxyuridine proliferation assay

Transfected LN229 and A1207 cells were plated in 6-well plates at 5×10³ cells/well and cultured for 24 h. After incubation with 2× EdU working solution (2 h, 37°C), cells were washed twice with PBS, fixed with 4% paraformaldehyde (30 min), and permeabilized with 2 mg/mL glycine and 0.5% Triton X-100. For detection, cells were stained with a 1:1 mixture of 1X Apollo and Hoechst 33342 (30 min). EdU-positive cells were quantified using fluorescence microscopy.

### Wound healing assay

The cells were transfected, then transferred to 6-well plates and allowed to grow to near-confluence. Linear scratches were generated in the cell monolayer via a sterile 200 μL pipette tip. After washing the wells with PBS, serum-free medium was added. Photographs of the wound areas were taken at 0 h and 48 h, and the variations in gap width were quantified with Image-J software.

### Transwell assay

Serum-starved cells (24 h) were suspended in Matrigel (BD Biosciences, USA) and plated in transwell upper chambers, with serum-containing medium in the lower chambers as chemoattractant. After 48 h, cells were fixed with 4% paraformaldehyde and stained with crystal violet for quantification.

### Prognostic risk scoring system for patient stratification

Based on clinical data obtained from the TCGA database, we initially identified 6 risk genes through univariate Cox regression analysis (63–66). To optimize model performance, LASSO (67–69) and multivariate Cox regression analyses were performed, ultimately selecting 5 key genes with independent prognostic value. A risk score model was constructed using the following formula: $\text{Risk Score}=\sum_{i}^{n}\text{Xi}\times \text{Yi}$ , where X represented the coefficient and Y denoted gene expression. Patients were stratified into the high and low risk groups based on the optimal cutoff value, and further evaluated through Kaplan-Meier and Receiver Operating Characteristic (ROC) curves (51, 70–72).

### Assessment of immunological profiles and pharmacological vulnerability

By applying the CIBERSORT algorithm, we first systematically analyzed the immune cell infiltration profiles of both groups. Based on these findings, we further calculated immune signature scores using the ESTIMATE. Additionally, to investigate potential differences in immune evasion between the two groups, we compared the Tumor Immune Dysfunction and Exclusion (TIDE) scores. Furthermore, to evaluate the differences in therapeutic drug sensitivity, we predicted the half-maximal inhibitory concentration (IC50) values of commonly used anticancer drugs using the “pRRophetic” package (v0.5) (73).

### Statistical analysis

For statistical analysis of the data, we employed R and Python. Differences between groups were assessed using the Wilcoxon rank-sum test, while Spearman’s correlation analysis was applied to examine associations between variables. All statistical tests were conducted as two-tailed tests, with a *P*-value <0.05 considered statistically significant, *P*-value < 0.01 considered highly significant, *P*-value < 0.001 considered very highly significant, and *P*-value < 0.0001 considered extremely significant.

## Results

### Single-cell transcriptomic profiling of GBM uncovered glioma cell heterogeneity

**Figure 1:**
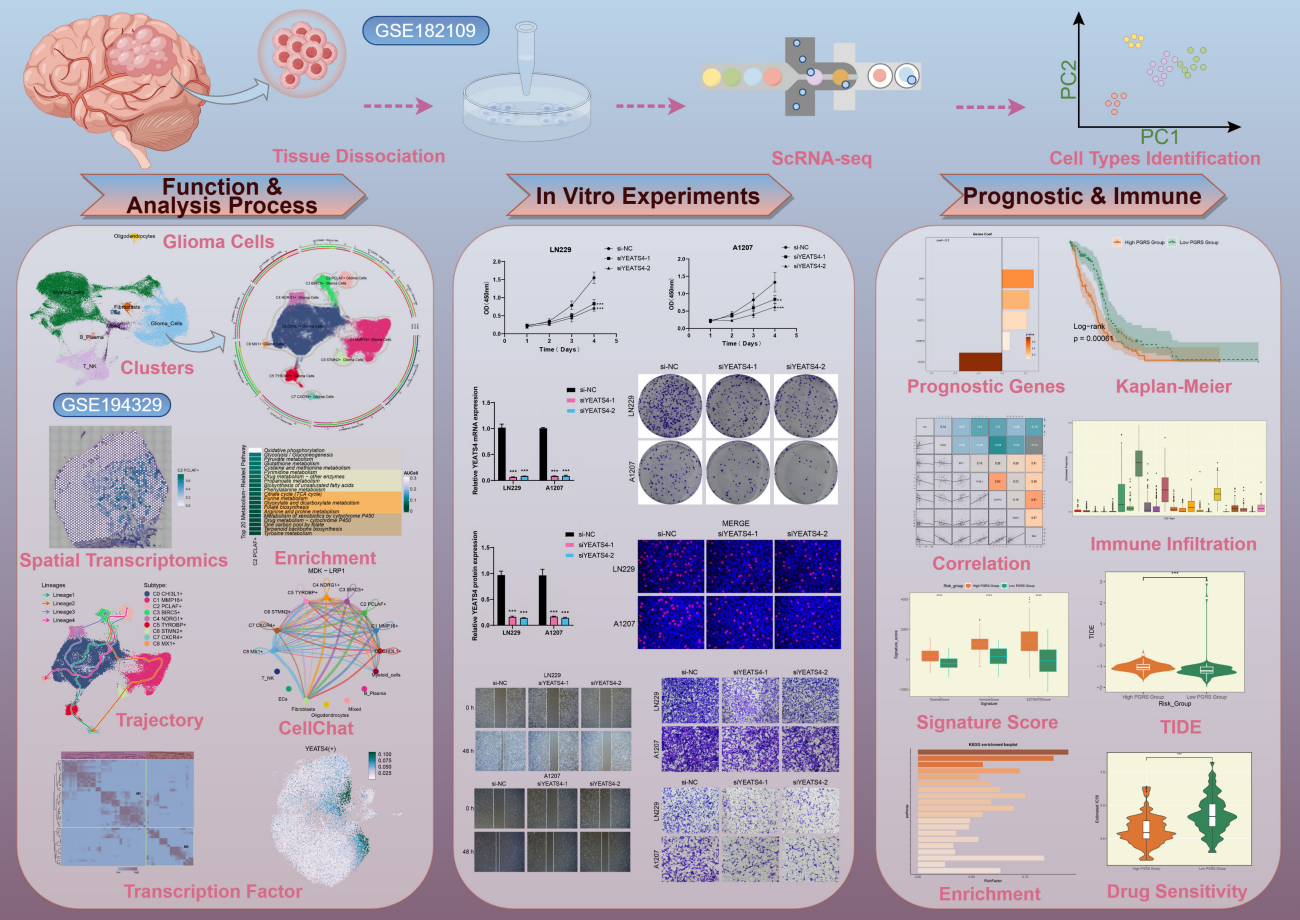
The workflow diagram for GBM scRNA-seq. The analysis steps for GBM scRNA-seq encompassed data preprocessing, characterization of key cell subtypes, and exploration of their biological significance.

**Figure 2:**
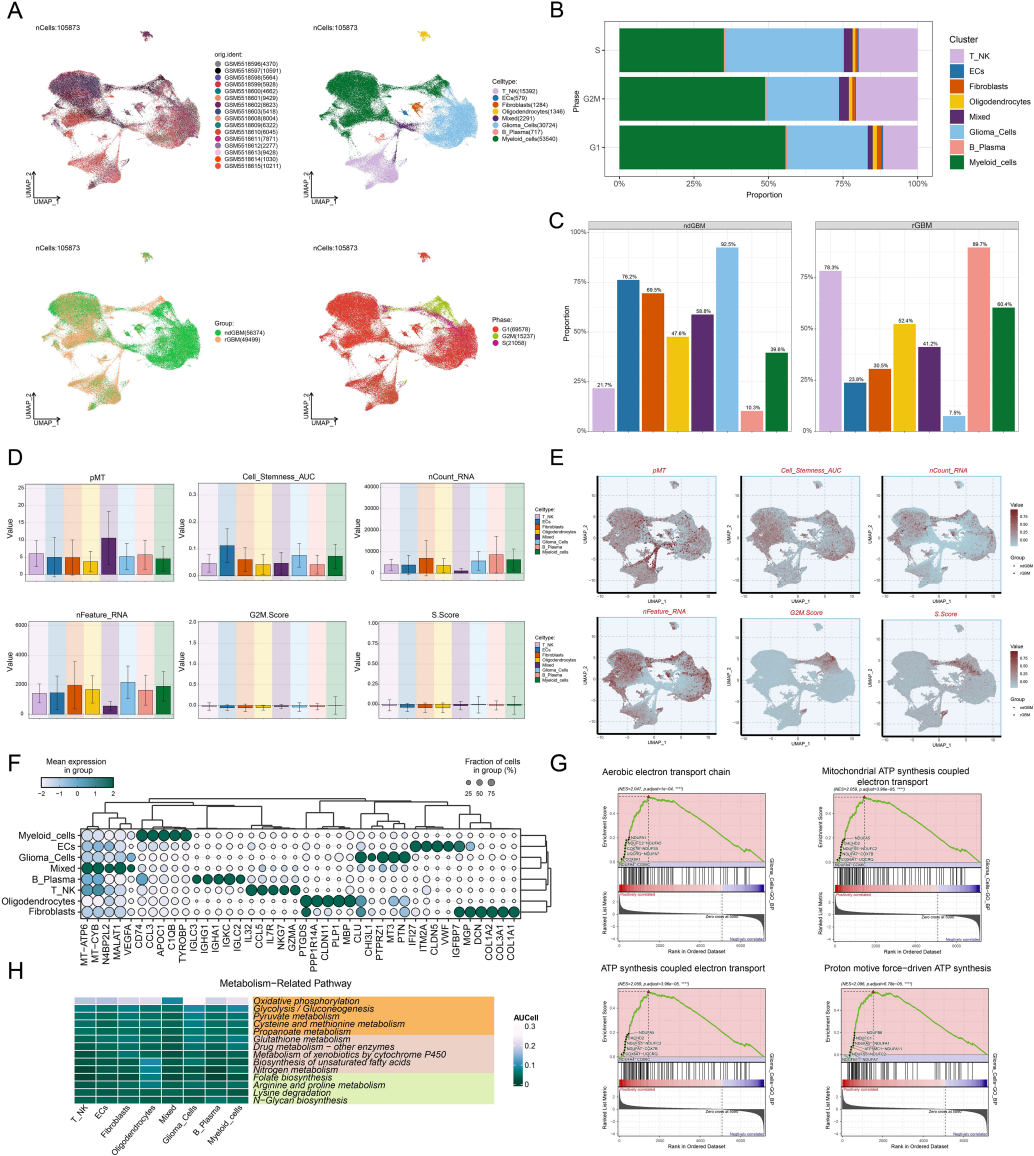
Comprehensive Single-Cell Characterization of GBM. **(A)** UMAP plots illustrated the distribution of 16 different samples (upper left), 8 cell types (upper right), various groups (lower left), and different cell cycle phases (lower right) across the entire cell population. **(B)** The stacked bar chart displayed the proportional distribution of each cell type across different cell cycle phases. **(C)** Distribution of different cell types across the two groups in proportion. **(D, E)** Bar plots and UMAP plots respectively illustrated the expression and distribution of pMT, Cell-Stemness-AUC, nCount-RNA, nFeature-RNA, G2/M.Score, and S.Score across different cell types. **(F)** The bubble plot demonstrated the mean expression levels of the top 5 DEGs in each cell type. **(G)** GSEA described the enrichment scores of biological processes associated with glioma cells. ****P < 0.0001. **(H)** Heatmap assessed the AUCellscores of the top metabolism-related pathways across 8 cell types.

We performed a comprehensive bioinformatics analysis of the obtained GBM dataset, with the complete analytical workflow spanning from raw data quality control to final visualization as illustrated in **Figure 1**. First, we systematically analyzed 16 samples from this dataset. Through rigorous cell quality filtering and standardized procedures, we ultimately obtained 105,873 high-quality cells for subsequent investigations. As illustrated in **Figure 2A**, the UMAP plots visualized the distribution characteristics of 16 samples and 8 cell types (T cells and NK cells, endothelial cells (ECs), fibroblasts, oligodendrocytes, mixed, glioma cells, B-plasma cells, and myeloid cells), while revealing the distribution characteristics between 2 groups (ndGBM and rGBM) and among 3 cell cycle phases (G1, G2/M, and S). Notably, through in-depth analysis of cell cycle and tissue origin across the 8 cell types, we observed a prominent feature: glioma cells showed significantly higher distribution proportion in S phase compared to G1 and G2/M phases (**Figure 2B**), with these cells predominantly originating from the ndGBM (**Figure 2C**). Further characterization revealed that glioma cells exhibited relatively high levels in multiple biological indicators, including pMT, Cell-Stemness-AUC, nCount-RNA, nFeature-RNA, G2/M.Score, and S.Score (**Figures 2D, E**). **Figure 2F** displayed the mean expression levels of the top 5 DEGs in each cell type. Of particular note, the signature genes of glioma cells included *CLU*, *CHI3L1*, *PTPRZ1*, *MT3*, and *PTN*, which may be closely associated with their malignant phenotype. Functional enrichment analysis demonstrated that biological processes related to glioma cells were primarily concentrated in energy metabolism pathways, including aerobic electron transport chain, mitochondrial ATP synthesis coupled electron transport, ATP synthesis coupled electron transport, and proton motive force-driven ATP synthesis (**Figure 2G**). Finally, we quantified metabolic pathway activities using the AUCell scores. The results identified oxidative phosphorylation, glycolysis/gluconeogenesis, pyruvate metabolism, cysteine and methionine metabolism, and propanoate metabolism as the top 5 most active metabolic pathways among the 8 cell types (**Figure 2H**). These findings demonstrated that glioma cells possessed high heterogeneity, proliferative activity, and metabolic characteristics, which was consistent with previously reported mechanisms whereby glioma cells drive tumor heterogeneity through metabolic abnormalities and reactive oxygen species accumulation (74). Based on these findings, we further systematically characterized the subtypes of glioma cells to elucidate the intrinsic relationship between their molecular heterogeneity and clinical phenotypes.

### Identification and multi-omics characterization of the C2 *PCLAF*+ subtype

**Figure 3:**
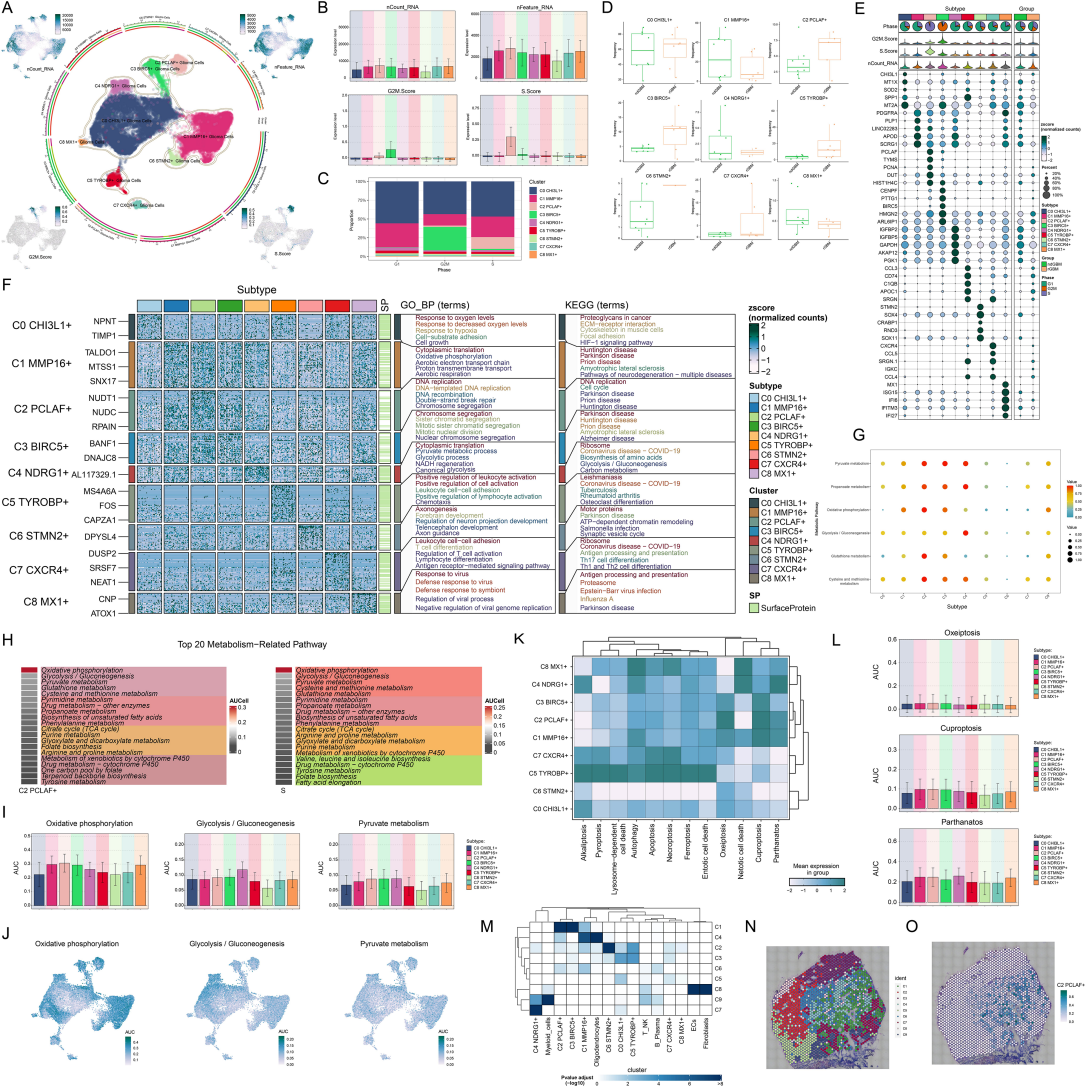
Molecular characterization and spatial distribution of the C2 *PCLAF*+ subtype. **(A)** The central part of the circular plot outlined the clustering of 9 glioma cell subtypes using contour lines, while the outer, middle, and inner axes displayed the logarithmic scale of clusters for each glioma cell subtype, as well as the distribution of groups and phases. The peripheral UMAP plots illustrated the distribution of nCount-RNA (upper left), nFeature-RNA (upper right), G2/M.Score (lower left), and S.Score (lower right) within glioma cells. **(B)** Bar plots visualized the expression levels of nCount-RNA, nFeature-RNA, G2/M.Score, and S.Score in each glioma cell subtype. **(C)** The stacked bar chart displayed the proportional distribution of each glioma cell subtype across different cell cycle phases. **(D)** Box plots illustrated the frequency of 9 glioma cell subtypes within each group. **(E)** The bubble plot displayed the top 5 marker genes in each glioma cell subtype. The pie charts showed the proportions of different phases, while the violin plots illustrated the expression levels of G2/M.Score, S.Score, and nCount-RNA. **(F)** Heatmap showed the enrichment analysis results of GB-BP and KEGG for 9 glioma cell subtypes. **(G)** The bubble plot illustrated the metabolic pathways in each glioma cell subtype. **(H)** The heatmaps assessed the AUCell scores of the top 20 metabolism-related pathways across the C2 *PCLAF*+ subtype and S phase. **(I, J)** Bar graphs and UMAP plots, based on the AUCell algorithm, respectively evaluated the activity and distribution of oxidative phosphorylation, glycolysis/gluconeogenesis, and pyruvate metabolism across different glioma cell subtypes. **(K)** Heatmap displayed the mean expression levels of programmed cell death pathways in each glioma cell subtype. **(L)** The AUCell algorithm was used to calculate the activities of oxeiptosis, cuproptosis, and parthanatos in each glioma cell subtype. **(M)** Heatmap displayed the enrichment degree of 9 spatial clusters associated with various cell types on ST 1 slide, as assessed by MIA analysis. **(N)** The visualization results of the spatial spots corresponding to the 9 spatial clusters were presented. **(O)** ST feature map illustrated the specific spatial expression pattern of the C2 *PCLAF*+ subtype.

Given the critical role of glioma cellular heterogeneity in GBM pathogenesis and treatment resistance, we systematically characterized their molecular features. Based on marker gene expression patterns, we identified 9 distinct glioma cell subtypes (**Figure 3A**), including C0 *CHI3L1*+ glioma cells, C1 *MMP16*+ glioma cells, C2 *PCLAF*+ glioma cells, C3 *BIRC5*+ glioma cells, C4 *NDRG1*+ glioma cells, C5 *TYROBP*+ glioma cells, C6 *STMN2*+ glioma cells, C7 *CXCR4*+ glioma cells, and C8 *MX1*+ glioma cells. UMAP visualization revealed significant transcriptomic differences and unique molecular characteristics among these subtypes. To further characterize the biological properties of these subtypes, we systematically analyzed their transcriptional activity and cell cycle features. The results demonstrated that the C2 *PCLAF*+ subtype exhibited significantly higher expression levels of nCount-RNA, nFeature-RNA, G2/M.Score, and S.Score compared to other subtypes, particularly in S.Score (**Figure 3B**). These findings suggested that this subtype likely possessed stronger proliferative activity. Through in-depth analysis of cell cycle distribution and tissue origin across different subtypes (**Figures 3C, D**), we observed that the C2 *PCLAF*+ subtype showed remarkably higher proportion in S phase and was predominantly derived from rGBM, characteristics that were highly consistent with its hyperproliferative phenotype. To elucidate the molecular characteristics of each subtype, we presented the top 5 marker genes for each subtype in **Figure 3E**. The C2 *PCLAF*+ subtype was characterized by *PCLAF*, *TYMS*, *PCNA*, *DUT*, and *HIST1H4C* as its top marker genes.

To further investigate the biological characteristics of different glioma cell subtypes, we conducted functional enrichment analysis (**Figure 3F**). The results indicated that the C0 *CHI3L1*+ subtype was mainly enriched in biological processes such as response to oxygen levels, response to decreased oxygen levels, response to hypoxia, cell-substrate adhesion, and cell growth, and was associated with pathways including proteoglycans in cancer, ECM-receptor interaction, cytoskeleton in muscle cells, focal adhesion, and HIF-1 signaling pathway. The C1 *MMP16*+ subtype was primarily enriched in biological processes such as cytoplasmic translation, oxidative phosphorylation, aerobic electron transport chain, proton transmembrane transport, and aerobic respiration, and was related to amyotrophic lateral sclerosis and pathways of neurodegeneration-multiple diseases. The C2 *PCLAF*+ subtype was predominantly enriched in biological processes such as DNA replication, DNA-templated DNA replication, DNA recombination, double-strand break repair, and chromosome segregation, and was related to pathways such as DNA replication and cell cycle. The C3 *BIRC5*+ subtype was mainly enriched in biological processes such as chromosome segregation, sister chromatid segregation, mitotic sister chromatid segregation, mitotic nuclear division, and nuclear chromosome segregation, and was related to pathways such as amyotrophic lateral sclerosis and Alzheimer disease. The C4 *NDRG1*+ subtype was mainly enriched in biological processes such as cytoplasmic translation, pyruvate metabolic process, glycolytic process, NADH regeneration, and canonical glycolysis, and was related to pathways such as ribosome, biosynthesis of amino acids, glycolysis/gluconeogenesis, and carbon metabolism. The C5 *TYROBP*+ subtype was mainly enriched in biological processes such as positive regulation of leukocyte activation, positive regulation of cell activation, leukocyte cell-cell adhesion, positive regulation of lymphocyte activation, and chemotaxis. The C6 *STMN2*+ subtype was mainly enriched in biological processes such as axonogenesis, forebrain development, regulation of neuron projection development, telencephalon development, and axon guidance, and was related to pathways such as motor proteins, ATP-dependent chromatin remodeling, salmonella infection, and synaptic vesicle cycle. The C7 *CXCR4*+ subtype was mainly enriched in biological processes such as leukocyte cell-cell adhesion, T cell differentiation, regulation of T cell activation, lymphocyte differentiation, and antigen receptor-mediated signaling pathway, and was related to pathways such as ribosome, antigen processing and presentation, Th17 cell differentiation, and Th1 and Th2 cell differentiation. The C8 *MX1*+ subtype was mainly enriched in biological processes such as response to virus, defense response to virus, defense response to symbiont, regulation of viral process, and negative regulation of viral genome replication, and was related to pathways such as antigen processing and presentation, and proteasome. Regarding metabolic features, we noticed that the metabolic pathways of 3 glioma cell subtypes are quite distinct (**Figure 3G**). The C2 *PCLAF*+ subtype was primarily active in pathways such as pyruvate metabolism, propanoate metabolism, oxidative phosphorylation, glutathione metabolism, and cysteine and methionine metabolism. The C3 *BIRC5*+ subtype was predominantly enriched in pyruvate metabolism and propanoate metabolism, whereas the C4 *NDRG1*+ subtype showed significant enrichment in pyruvate metabolism, propanoate metabolism and glycolysis/gluconeogenesis. Quantitative comparison of metabolic pathway activities using AUCell scores (**Figure 3H**) revealed that the top 20 metabolic pathways of the C2 *PCLAF*+ subtype matched those of S phase. Further visualization analysis (**Figures 3I, J**) revealed that compared with other subtypes, the C2 *PCLAF*+ subtype showed significantly enhanced activity in 3 critical metabolic pathways: oxidative phosphorylation, glycolysis/gluconeogenesis, and pyruvate metabolism. Additionally, analysis of programmed cell death pathways (**Figures 3K, L**) demonstrated that this subtype showed relatively higher AUC scores in oxeiptosis, cuproptosis, and parthanatos pathways, but lower AUC scores in alkaliptosis and netotic cell death pathways. This distinctive expression pattern implied the existence of unique cell death regulation mechanisms in this subtype.

### STs revealed the spatial expression patterns of proliferative C2 *PCLAF*+ subtype

To further investigate the spatial heterogeneity of GBM, we selected two representative GBM sections for ST analysis. In ST 1 slide, MIA and TransferData analyses revealed that the C2 *PCLAF*+ subtype was predominantly enriched in spatial cluster C1 (**Figure 3M**). Comparative analysis of spatial distribution patterns demonstrated a high degree of similarity between the spatial localization pattern of cluster C1 (**Figure 3N**) and the distribution characteristics of the C2 *PCLAF*+ subtype (**Figure 3O**). To validate this finding, we employed RCTD analysis to map the spatial regions with high expression of the C2 *PCLAF*+ subtype (**Supplementary Figure 1A**), which showed significant spatial co-localization with regions exhibiting high *PCLAF* gene expression (**Supplementary Figure 1B**). In ST 2 slide, the spatial distribution patterns of nCount-Spatial, nFeature-Spatial, G2/M.Score, and S.Score were highly consistent with the characteristics of the C2 *PCLAF*+ subtype (**Supplementary Figure 1C**), further confirming its unique spatial organization within the GBM architecture.

### C2 *PCLAF*+ subtype-driven differentiation promoted GBM progression

**Figure 4:**
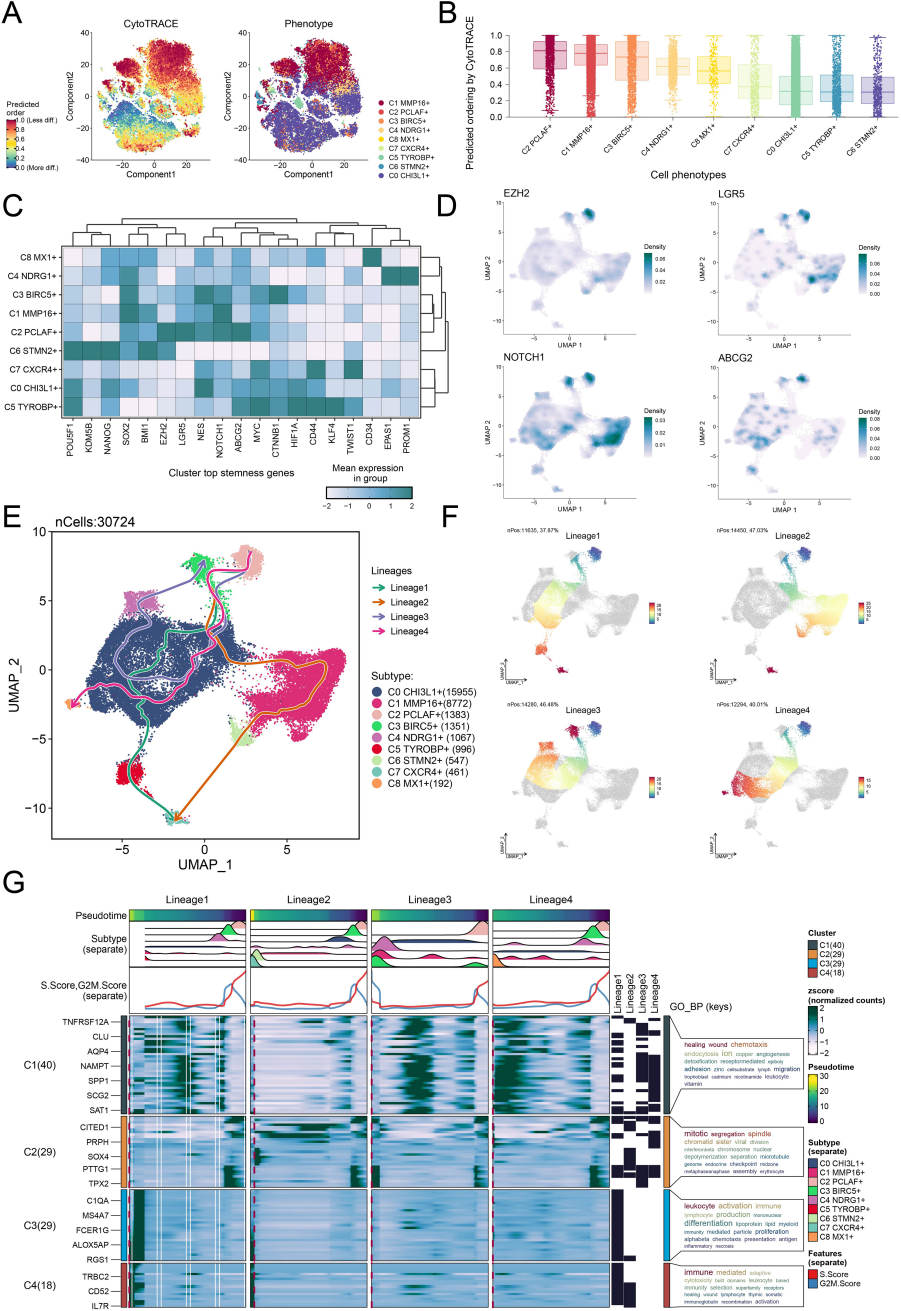
Differentiation dynamics and lineage characteristics of glioma cell subtypes. **(A)** The two-dimensional graphs utilized CytoTRACE to assess the predicted order (left) and distribution characteristics (right) of the 9 glioma cell subtypes. **(B)** Box plot demonstrated the ordering of the 9 glioma cell subtypes as predicted by the CytoTRACE analysis. **(C)** Heatmap displayed the mean expression of top stemness genes in the 9 cell clusters. **(D)** UMAP plots visualized the distribution of top stemness genes in the C2 *PCLAF*+ subtype. **(E)** The Slingshot analysis depicted 4 differentiation lineages of the 9 glioma cell subtypes, with the C2 *PCLAF*+ subtype appearing in the early stages of differentiation. **(F)** UMAP plots represented the progression of 4 lineages along the inferred pseudotime trajectories. **(G)** The GO-BP enrichment analysis results revealed key biological processes associated with the 4 lineages during glioma cell differentiation.

To probe the developmental origin of GBM and elucidate its heterogeneity, we systematically investigated the differentiation characteristics of glioma cell subtypes. We used CytoTRACE to predict the stemness ranking of 9 glioma cell subtypes and found that the C2 *PCLAF*+ subtype exhibited high stemness (**Figures 4A, B**). This result suggested that the C2 *PCLAF*+ subtype might possess stronger stem cell-like properties, laying the foundation for further investigation into its differentiation potential. We evaluated the mean expression of top stemness genes in each cell cluster and visualized the distribution of top stemness genes (*EZH2*, *LGR5*, *NOTCH1*, and *ABCG2*) in the C2 *PCLAF*+ subtype (**Figures 4C, D**). To comprehensively delineate the differentiation trajectories of glioma cells, we employed Slingshot to assess the differentiation lineages of the 9 glioma cell subtypes (**Figure 4E**). Notably, the analysis demonstrated that all 4 lineages originated from the C2 *PCLAF*+ subtype, further supporting its pivotal role as a differentiation initiator. Subsequently, we visualized the progression of lineage 1, lineage 2, lineage 3, and lineage 4 along the inferred pseudotemporal trajectories (**Figure 4F**), which provided more intuitive evidence for the central position of the C2 *PCLAF*+ subtype in the differentiation process and revealed distinct differentiation pathways among the lineages. Finally, to explore the biological characteristics of the different differentiation lineages, we further examined the major biological processes associated with the 4 lineages during glioma cell differentiation (**Figure 4G**). Functional enrichment analysis revealed that cluster C1 was primarily enriched in healing wound and chemotaxis processes, cluster C2 was mainly associated with mitotic segregation and spindle-related functions, cluster C3 showed strong correlations with leukocyte activation and immune responses, while cluster C4 was significantly linked to immune-mediated and adaptive mechanisms.

### Interplay between the C2 *PCLAF*+ subtype and fibroblasts through the MDK-LRP1 ligand–receptor pair

**Figure 5:**
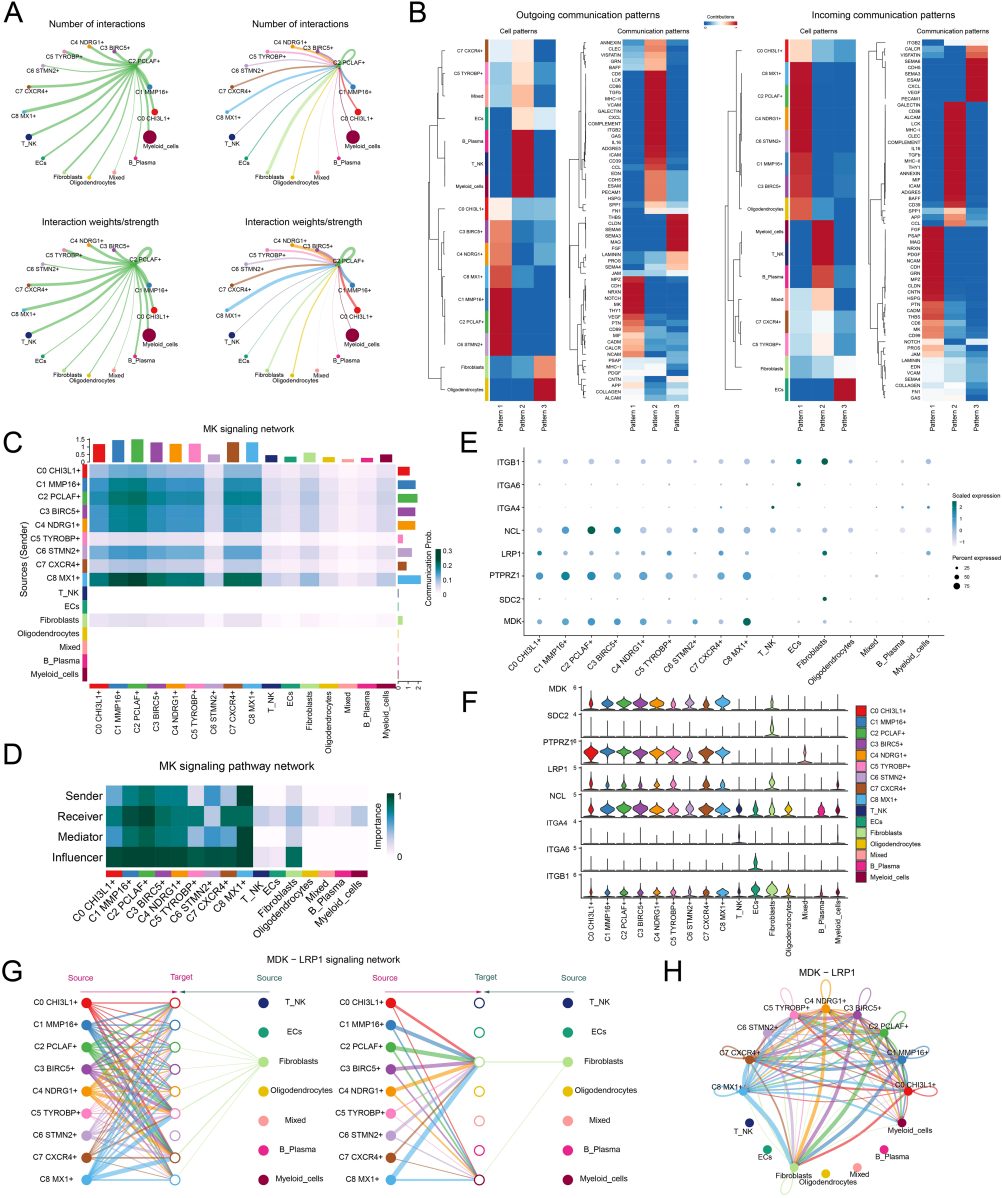
Single-cell communication atlas dominated by the C2 *PCLAF*+ subtype. **(A)** Circle diagrams illustrated the interactions of the C2 *PCLAF*+ subtype, presenting it as a source (left) and a target (right) in relation to other cell types, highlighting both the number (upper) and the intensity (lower) of these interactions. **(B)** Heatmaps displayed the outgoing (left) and incoming (right) communication patterns of 9 glioma cell subtypes and 7 other cell types under 3 cell communication patterns, as well as the key proteins involved in these 3 communication patterns. **(C)** Heatmap depicted the communication probabilities of 9 glioma cell subtypes and 7 other cell types under the MK signaling network. **(D)** Heatmap illustrated the importance of 9 glioma cell subtypes and 7 other cell types within the MK signaling pathway network, including sender, receiver, mediator, and influencer. **(E, F)** The bubble plot and violin plots visualized the expression patterns of essential ligands and receptors within the MK signaling pathway, encompassing 9 glioma cell subtypes and 7 additional cell types. **(G, H)** The hierarchical graph and circle diagram depicted the interactions among 9 glioma cell subtypes and 7 other cell types within the MDK-LRP1 ligand–receptor pair.

Building upon the pivotal role of the C2 *PCLAF*+ subtype in GBM differentiation, we deeply analyzed its molecular mechanisms in regulating microenvironmental communication. First, we quantitatively assessed the number and intensity of interactions between the C2 *PCLAF*+ subtype and all other cell types, revealing enhanced communication between this subtype and fibroblasts (**Figure 5A**). To comprehensively characterize the intercellular communication network, we further analyzed the outgoing and incoming communication patterns among 9 glioma cell subtypes and 7 other cell types (**Figure 5B**). Notably, the analysis demonstrated that both outgoing and incoming signals of the C2 *PCLAF*+ subtype were predominantly regulated by Pattern 1, with significant contributions from key molecules including MPZ, CDH, NRXN, NCAM, PTN, MK, and CD99. These molecules likely collectively formed the molecular basis of C2 *PCLAF*+ subtype communication. Of particular note, our analysis of communication probabilities within the MK signaling network (**Figure 5C**) showed increased communication likelihood between the C2 *PCLAF*+ subtype and fibroblasts. To elucidate the mechanism underlying this specific communication, we performed in-depth analysis of functional roles for various cell types within the MK signaling pathway network (**Figure 5D**). The results indicated that the C2 *PCLAF*+ subtype exhibited multifaceted communication functions, primarily serving as sender, receiver, mediator, and influencer, while fibroblasts mainly participated as receivers and influencers in the communication network. Additionally, through visual analysis, we found that the key ligand MDK and its receptor LRP1 in the MK signaling pathway were highly expressed in both the C2 *PCLAF*+ subtype and fibroblasts (**Figures 5E, F**). Based on this, we further focused on the MDK-LRP1 signaling axis and confirmed significant cellular interactions between the C2 *PCLAF*+ subtype and fibroblasts (**Figures 5G, H**).

### TF activity mapping identified proliferation-driving characteristics in C2 *PCLAF*+ subtype

**Figure 6:**
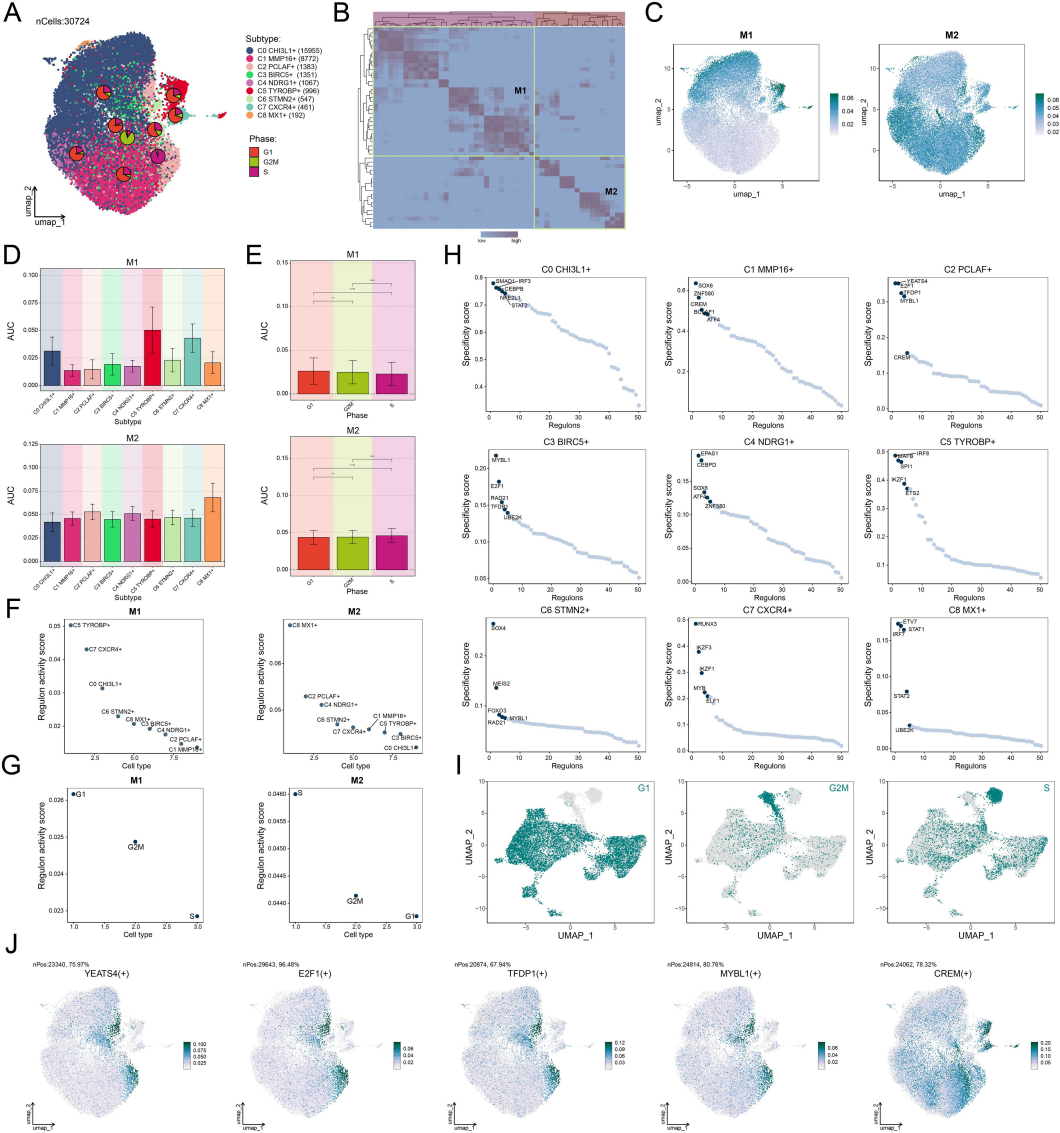
Decoding the transcriptional regulatory network of the C2 *PCLAF*+ subtype. **(A)** UMAP plots displayed the distribution of clustering for 9 glioma cell subtypes based on regulon activity level, and the pie charts illustrated the proportions of their phases. **(B)** Heatmap revealed two regulatory modules (M1 and M2) identified based on TF recognition in glioma cell subtypes. **(C)** UMAP plots showed the distribution of TFs expression in M1 and M2. **(D, E)** Bar plots visualized the AUC values of different glioma cell subtypes and phases within M1 and M2. **P < 0.01, ****P < 0.0001. **(F, G)** Scatter plots exhibited the ranking of regulon activity scores for various glioma cell subtypes and phases within M1 and M2. **(H)** Scatter plots presented the ranking of specificity scores for the top 5 TFs in each subtype of glioma cells. **(I)** UMAP plots visualized the expression distribution of TFs in the G1, G2/M, and S phases. **(J)** UMAP plots visualized the expression distribution of the top 5 TFs in the C2 *PCLAF*+ subtype within M2.

To elucidate the molecular characteristics of different glioma cell subtypes, we performed a re-clustering analysis of glioma cells based on regulon activity and integrated the cell cycle phases. The results revealed that the C2 *PCLAF*+ subtype represented a large proportion in the S phase (**Figure 6A**), suggesting its high DNA replication activity and potential association with tumor proliferation. To further explore the TF network regulating the C2 *PCLAF*+ subtype, we divided functionally and expression-similar TFs into two regulatory modules, M1 and M2, based on AUCell similarity scores (**Figure 6B**). UMAP visualization demonstrated distinct distribution patterns of TFs in M1 and M2 across glioma cells (**Figure 6C**). Notably, the C2 *PCLAF*+ subtype exhibited higher expression in M2 (**Figure 6D**), and S-phase cells within M2 showed elevated TF expression (**Figure 6E**). Further analysis revealed that both the C2 *PCLAF*+ subtype and S-phase cells displayed relatively higher regulon activity scores in the M2 (**Figures 6F, G**), further supporting the link between this subtype and proliferative activity. To decipher the core regulatory mechanisms of the C2 *PCLAF*+ subtype, we identified the top 5 TFs with the highest specificity scores in each subtype (**Figure 6H**). In the C2 *PCLAF*+ subtype, the top 5 TFs were YEATS4, E2F1, TFDP1, MYBL1, and CREM, indicating their potential cooperative role in driving the proliferative phenotype of this subtype. Ultimately, we delineated the phase-specific distribution characteristics of TFs during G1, G2/M and S phases using UMAP (**Figure 6I**) and specifically highlighted the distribution patterns of the top 5 TFs in the C2 *PCLAF*+ subtype within the M2 module (**Figure 6J**). We observed significantly higher expression of YEATS4 in the C2 *PCLAF*+ subtype, prompting further *in vitro* experiments to validate the functional role of YEATS4 in GBM progression.

### *In vitro* experimental validation of the functional characterization of YEATS4 in GBM progression

**Figure 7:**
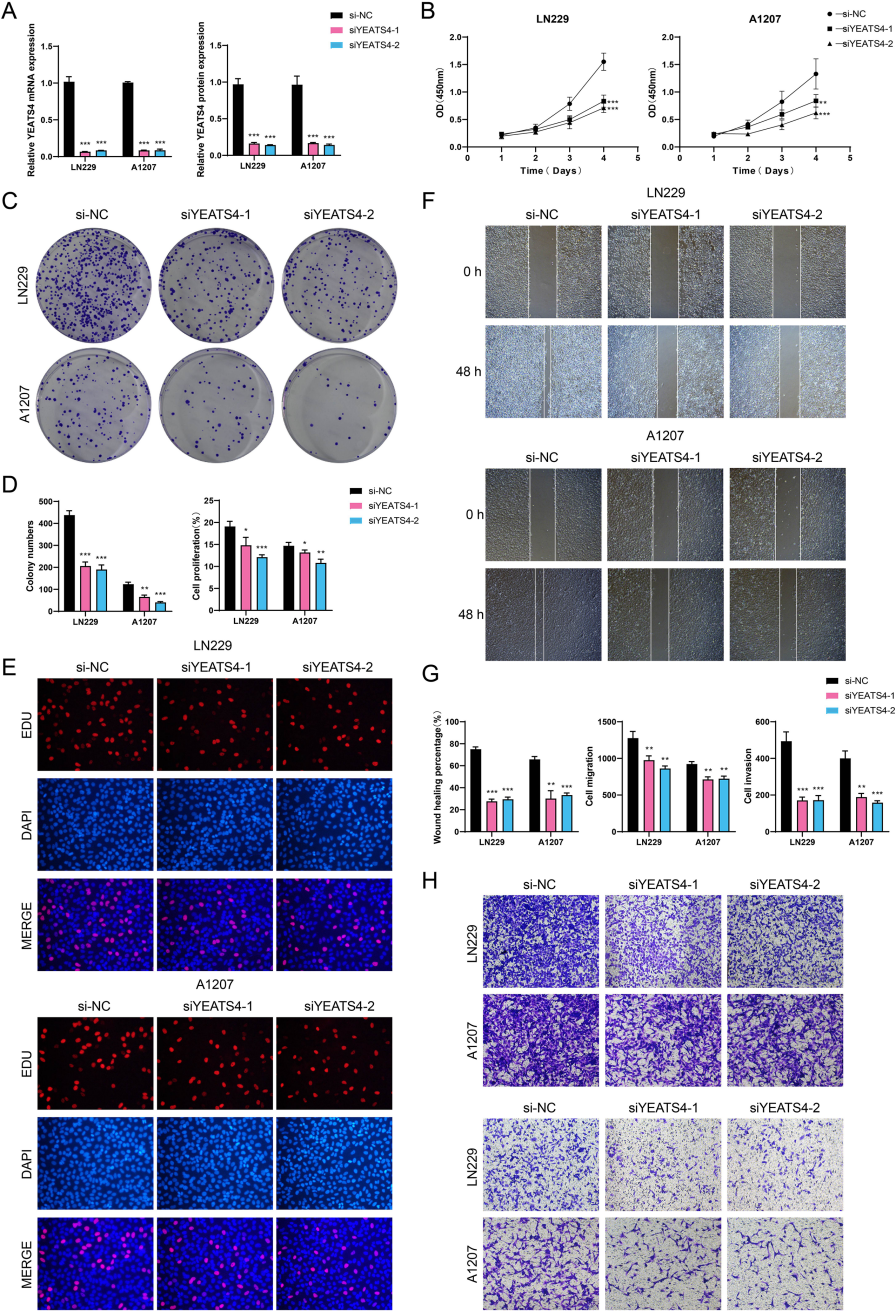
*In vitro* experimental verification. **(A)** Bar plots presented the relative expression levels of YEATS4 mRNA and protein in LN229 and A1207 GBM cell lines. si-NC, si*YEATS4*-1, and si*YEATS4–*2 represented different siRNA treatment groups, which were used to compare the effects of *YEATS4* knockdown on expression levels. **(B)** The line graphs illustrated the cell viability over time (0 to 4 days) in two GBM cell lines treated with si-NC, si*YEATS4*-1, and si*YEATS4*-2. **(C)** The colony formation assay was conducted to compare the effects of si-NC, si*YEATS4*-1, and si*YEATS4–*2 on colony formation in two GBM cell lines. **(D)** Bar plots illustrated the colony numbers and cell proliferation in two GBM cell lines treated with si-NC, si*YEATS4*-1, and si*YEATS4*-2. **(E)** The EDU staining assay was performed to assess cell proliferation in two GBM cell lines treated with si-NC, si*YEATS4*-1, and si*YEATS4*-2. **(F)** The cell wound healing assay evaluated the wound healing ability of two GBM cell lines at 0 h and 48 h following treatment with si-NC, si*YEATS4*-1, and si*YEATS4*-2. **(G)** Bar plots illustrated the wound healing percentage, cell migration, and cell invasion in two GBM cell lines treated with si-NC, si*YEATS4*-1, and si*YEATS4*-2. **(H)** Transwell assay evaluated the cell migration (upper) and invasion capabilities (lower) of two GBM cell lines treated with si-NC, si*YEATS4*-1, and si*YEATS4*-2. *P < 0.05, **P < 0.01, ***P < 0.001.

We conducted *in vitro* experiments using LN229 and A1207 GBM cell lines to investigate the functional consequences of *YEATS4* knockdown. These two cell lines were divided into 3 treatment groups: si-NC (negative control), si*YEATS4*-1, and si*YEATS4*-2. As shown in **Figure 7A**, the mRNA and protein expression levels of YEATS4 were significantly reduced following knockdown. We monitored the average optical density values of the 2 GBM cell lines treated with si-NC, si*YEATS4*-1, and si*YEATS4–*2 over time (0 to 4 days) and found that *YEATS4* knockdown significantly inhibited cell viability (**Figure 7B**). Subsequently, colony formation assays revealed a marked reduction in the number of colonies after *YEATS4* knockdown (**Figures 7C, D**). Aligned with these discoveries, EDU staining confirmed that cell proliferation was impaired following *YEATS4* knockdown (**Figures 7D, E**). Moreover, wound healing assays demonstrated significantly delayed wound closure at 48 h post-knockdown (**Figures 7F, G**). Most strikingly, transwell assays showed that the migratory and invasive capacities of both GBM cell lines were simultaneously impaired following *YEATS4* knockdown (**Figures 7G, H**).

### The construction and analytical validation of a prognostic risk model based on C2 *PCLAF*+ glioma cells

**Figure 8:**
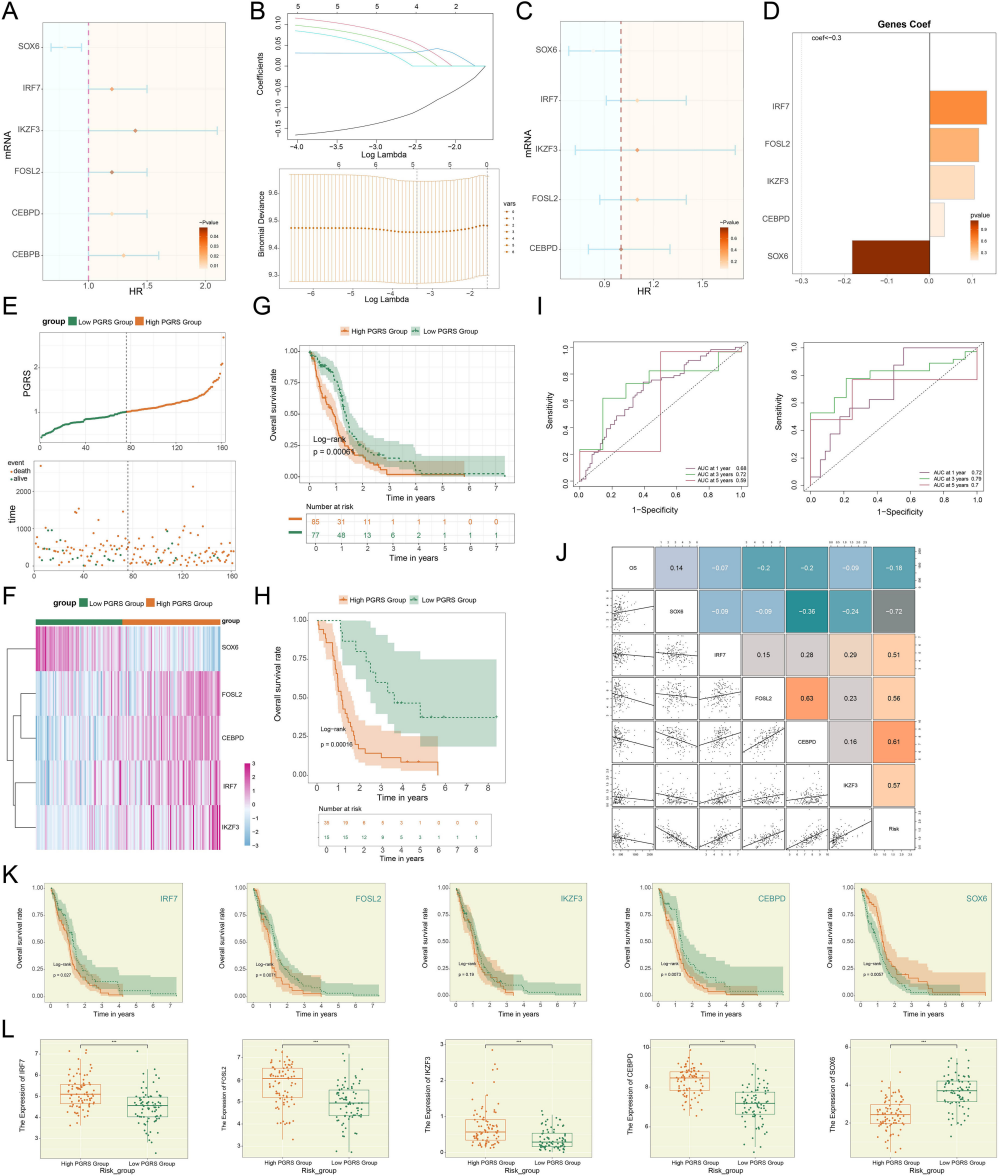
Development of a risk scoring model. **(A)** The forest plot demonstrated the results of univariate Cox regression analysis for key genes. Hazard ratios (HR) and their confidence intervals (lines) were used to evaluate prognostic associations: an HR < 1 indicated a protective factor, while an HR > 1 indicated a risk factor. **(B)** LASSO regression results. Optimal lambda selection via cross-validation (upper); Coefficient trajectories (upward for significant genes) at optimal lambda (lower). **(C)** The forest plot displayed the results of multivariate Cox regression analysis for key genes. **(D)** Bar plot compared the distribution of coefficients for the selected genes. **(E)** The survival curve combined with scatter plot revealed the survival rate trends over time in the high PGRS group (orange) and low PGRS group (green), with event endpoints including death and alive. **(F)** Heatmap demonstrated the comparative results of prognostic gene expression levels between high PGRS group and low PGRS group. **(G, H)** The Kaplan-Meier survival curves compared the OS rates between the high and low PGRS groups in the research (upper) and external cohorts (lower). The table at the bottom displayed the number at risk at each annual time point. **(I)** The ROC curves depicted the area AUC for 1-year, 3-year, and 5-year survival predictions in the research (left) and external cohorts (right). **(J)** The matrix heatmap combined with the scatter plot depicted the correlations between prognostic genes and both OS and risk scores. **(K)** The Kaplan-Meier survival curves compared the OS rates among patients stratified by expression levels of the 5 prognostic genes. **(L)** Box plots showed differential expression of the 5 prognostic genes between high and low PGRS groups. ***P < 0.001.

The prognosis assessment of GBM urgently required reliable molecular markers. To address this, we established a risk scoring model founded on C2 *PCLAF*+ glioma cells. In the process of constructing the model, we first preliminarily screened 6 risk genes closely tied to patient survival (**Figure 8A**). To further optimize the specificity of the model, LASSO regression analysis was employed to select candidate genes (**Figure 8B**), followed by multivariate Cox regression analysis, which ultimately identified 5 genes with independent prognostic value (**Figure 8C**). Notably, coefficient value analysis revealed that the positive coefficients of *IRF7*, *FOSL2*, *IKZF3*, and *CEBPD* indicated their high expression portended poorer patient outcomes (**Figure 8D**). To validate the clinical significance of these genes, patients were dichotomized by the *PCLAF*+ glioma cells risk score (PGRS) cutoff (**Figure 8E**). Comparative analysis demonstrated that significant upregulation of *IRF7*, *FOSL2*, *IKZF3*, and *CEBPD*, along with downregulation of *SOX6*, in the high PGRS group compared to controls (**Figure 8F**). This result was highly consistent with survival analysis: Kaplan-Meier curves showed that the OS rate was lower in the high PGRS group (**Figure 8G**), further supporting the prognostic value of PGRS stratification. Meanwhile, external datasets were used for validation, and the results showed that the two sets of data had good consistency (**Figure 8H**). The predictive performance of the model was validated using time-dependent ROC curves. As shown in **Figure 8I**, the model demonstrated high sensitivity and specificity in predicting 1-year, 3-year, and 5-year survival, with AUC values indicating reliable discriminative ability. More importantly, the successful validation of the external validation dataset has significantly enhanced the clinical applicability and generalization ability of the model (75). Follow-up studies assessed connections between prognostic genes and clinical outcomes: *IRF7*, *FOSL2*, *IKZF3*, and *CEBPD* were not only significantly negatively correlated with OS but also positively correlated with risk scores, whereas *SOX6* exhibited the opposite pattern—its high expression was associated with prolonged OS and reduced risk scores (**Figure 8J**). This association was further confirmed by gene expression stratification analysis: as shown in **Figure 8K**, patients with high expression of *IRF7*, *FOSL2*, and *CEBPD* had significantly reduced OS rates, while those with high *SOX6* expression showed survival advantages. Finally, we observed significant differences in the expression patterns of these 5 prognostic genes between the two groups (**Figure 8L**): *IRF7*, *FOSL2*, *IKZF3*, and *CEBPD* were generally highly expressed in the high PGRS group, whereas *SOX6* was specifically enriched in the low PGRS group.

### The PGRS risk scoring system revealed GBM immune microenvironment characteristics and predicted immunotherapy sensitivity

**Figure 9:**
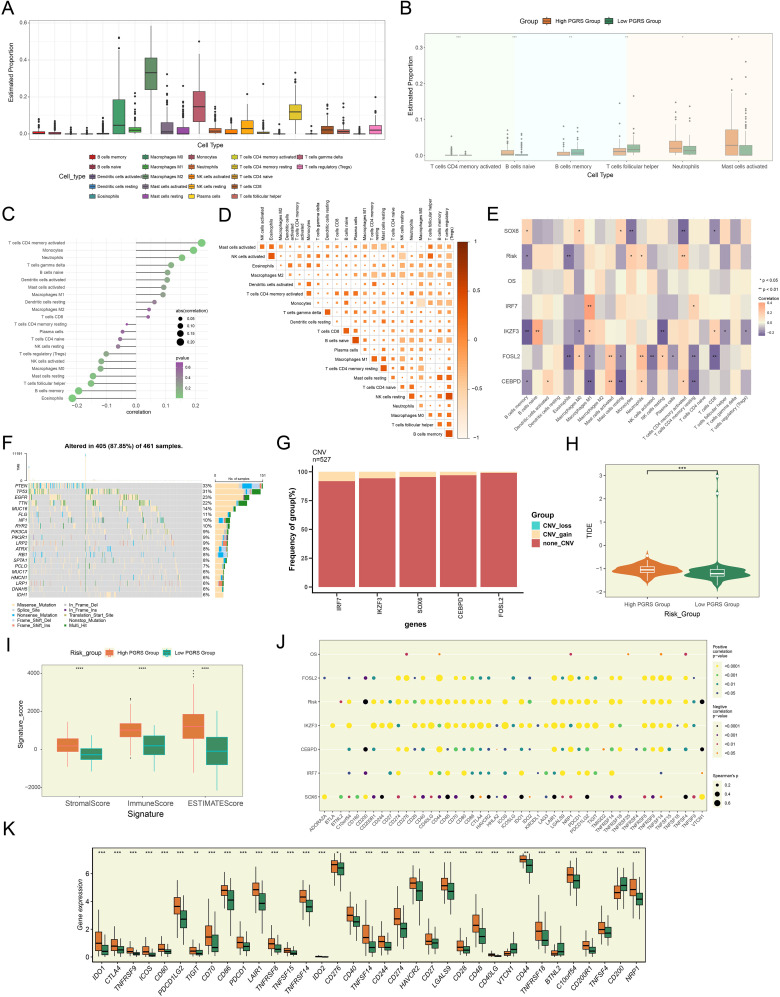
The pivotal role of PGRS scoring in the regulation of the GBM immune network. **(A)** Box plot demonstrated the immune cell infiltration landscape. **(B)** Box plot revealed the proportions of immune cell infiltration between high and low PGRS groups. **(C)** The lollipop plot depicted the association analysis between different immune cell types and risk scores. **(D)** The matrix heatmap displayed pairwise correlation strengths between different immune cell types. **(E)** Heatmap showcased correlation strengths between 5 prognostic genes, OS, risk scores, and various immune cell types. **(F)** The waterfall plot integrated with bar charts displayed the mutation frequencies and variant types of the top 20 mutated genes. **(G)** The stacked bar plot presented the frequency analysis of CNV for 5 prognostic genes. **(H)** Box plot combined with violin plot compared TIDE scores between high and low PGRS groups. **(I)** Box plot illustrated the stromal, immune, and ESTIMATE scores between high and low PGRS groups. **(J)** The bubble plot revealed the correlation analysis between 5 prognostic genes, OS, risk scores, and immune checkpoint-related genes. **(K)** Box plot highlighted differential expression of immune checkpoint-related genes between high and low PGRS groups. *P < 0.05, **P < 0.01, ***P < 0.001, and ****P < 0.0001.

Building upon our previously established PGRS scoring system, we proceeded to explore its immunomodulatory implications. We noted significant heterogeneity in the infiltration proportions of various immune cell types in **Figure 9A**, which prompted us to explore their relationship with the PGRS score. In-depth analysis revealed that B cells naive, Neutrophils, and Mast cells activated were significantly enriched in the high PGRS group, while B cells memory and T cells follicular helper were predominant in the low PGRS group (**Figure 9B**). Notably, this distribution pattern was consistent with the correlation analysis of risk scores: T cells CD4 memory activated, Monocytes, and Neutrophils showed significant positive correlations, whereas T cells follicular helper, B cells memory, and Eosinophils exhibited clear negative trends (**Figure 9C**). Collective evidence collectively suggested that the PGRS score was intimately related to the infiltration characteristics of specific immune cell types. Further examination of the immune microenvironment features revealed a robust interaction network among different immune cell types, with generally high pairwise correlation strengths (**Figure 9D**). More importantly, the 5 key prognostic genes demonstrated significant correlation patterns with multiple immune cell types (**Figure 9E**), among which the strong positive correlations between *IRF7* and Macrophages M1, as well as *IKZF3* and B cells naive, were particularly prominent. These molecular-immune associations provided new insights into the regulatory mechanisms of prognostic genes. In the genomic feature analysis, we systematically evaluated the mutation profiles of 461 samples and found somatic mutations in 405 (87.85%) cases, with missense mutations being the predominant type. Notably, *PTEN*, *TP53*, and *EGFR* exhibited relatively high mutation frequencies (**Figure 9F**). The CNV analysis indicated the CNV gains in the 5 prognostic genes, particularly prominent for *IRF7* and *IKZF3* (**Figure 9G**). To comprehensively assess the potential for immunotherapy response, we compared TIDE and signature scores between high and low PGRS groups. The results showed that the high PGRS group had higher TIDE, stromal, immune, and ESTIMATE scores (**Figures 9H, I**). This difference was further corroborated in the immune checkpoint analysis: most immune checkpoint-related genes not only showed positive correlations with risk scores but also exhibited significant co-upregulation with *IRF7*, *FOSL2*, *IKZF3*, and *CEBPD*, while only *SOX6* displayed a negative correlation pattern (**Figure 9J**). Gene expression profiling confirmed that most immune checkpoint-related genes, such as *CD276*, *CD44*, and *C10orf54*, were significantly upregulated in the high PGRS group (**Figure 9K**). These results jointly pointed to increased immune checkpoint inhibitor susceptibility in the high PGRS group.

### Functional enrichment characteristics of DEGs and drug sensitivity analysis

**Figure 10:**
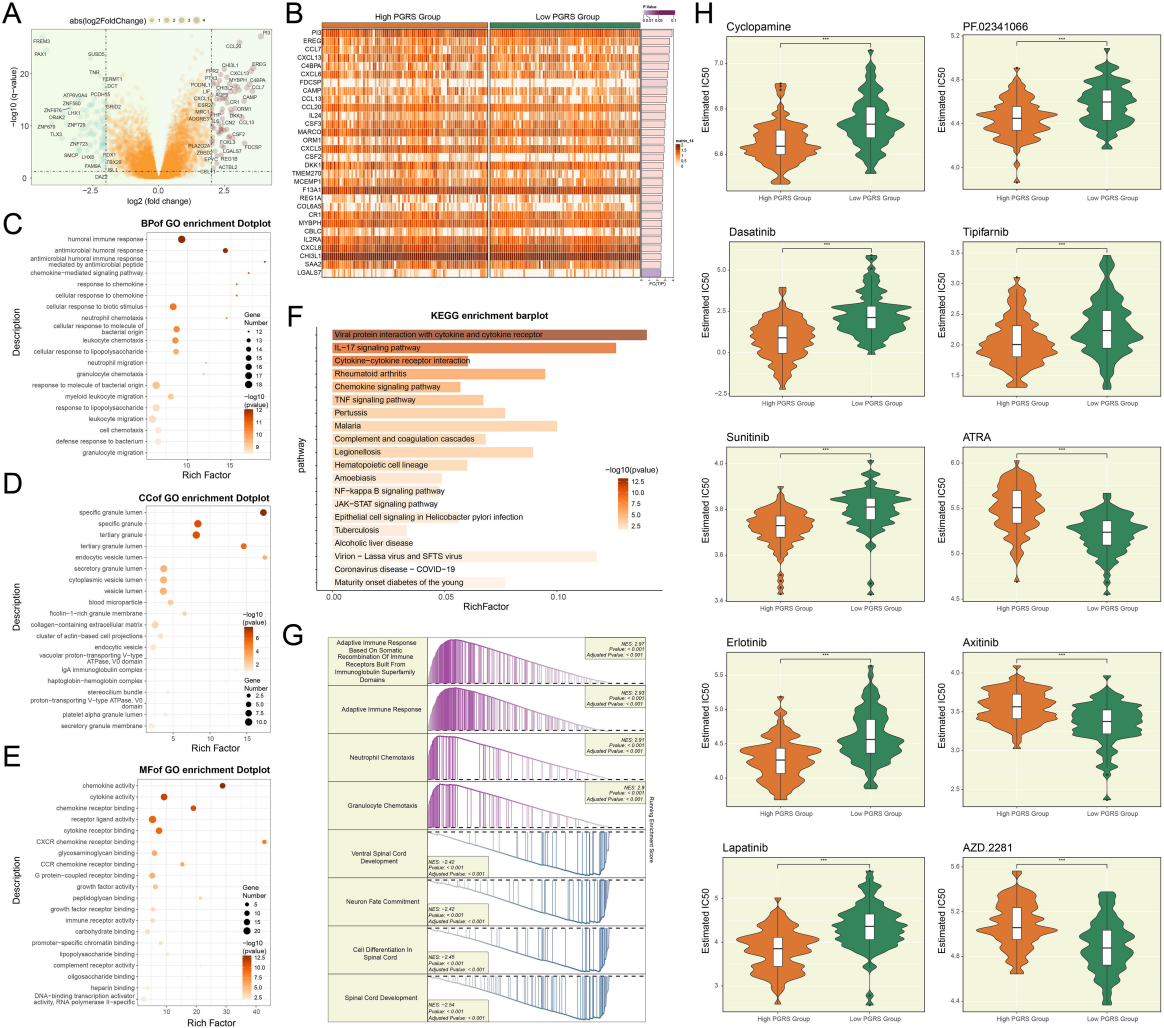
Functional enrichment and drug response in PGRS-stratified groups. **(A)** The volcano plot highlighted the significantly upregulated or downregulated genes between high and low PGRS groups. **(B)** Heatmap illustrated the DEGs expression levels between high and low PGRS group. **(C–E)** GO enrichment analysis of DEGs across biological processes, cellular components, and molecular functions. **(F)** KEGG enrichment analysis of DEGs revealed significantly enriched biological pathways. **(G)** GSEA enrichment analysis of DEGs showed significantly enriched biological processes, with positive and negative normalized enrichment scores (NES) indicating directional activation or suppression. **(H)** Box plots combined with violin plots evaluated the IC50 values of different drugs between high and low PGRS groups. ***P < 0.001.

To delineate the molecular characteristic distinctions across PGRS stratification groups and their potential clinical significance, we comprehensively analyzed the DEGs, functional pathway characteristics, and drug sensitivity differences between the two groups. In **Figure 10A**, we displayed the significantly upregulated or downregulated genes between the high and low PGRS groups and further visualized their expression patterns (**Figure 10B**). Subsequently, we performed systematic functional annotation analysis on these DEGs. Biological processes analysis revealed that the DEGs were highly abundant in humoral immune response, antimicrobial humoral response, and antimicrobial humoral immune response mediated by antimicrobial peptide (**Figure 10C**). Regarding cellular components, the DEGs were primarily associated with structures such as specific granule lumen, specific granule and tertiary granule (**Figure 10D**). Molecular functions analysis indicated that these genes were significantly enriched in chemokine activity, cytokine activity and chemokine receptor binding (**Figure 10E**). KEGG enrichment analysis further supported these findings, showing that the DEGs were significantly enriched in pathways including viral protein interaction with cytokine and cytokine receptor, and IL-17 signaling pathway (**Figure 10F**). To further elucidate the biological significance of the DEGs, we conducted GSEA enrichment analysis, which demonstrated that these genes were significantly involved in key biological processes such as adaptive immune response based on somatic recombination of immune receptors built from immunoglobulin superfamily domains, adaptive immune response, neutrophil chemotaxis, and granulocyte chemotaxis (**Figure 10G**). Finally, the drug sensitivity results disclosed that the high PGRS group exhibited lower IC50 values for Cyclopamine, Dasatinib, Sunitinib, Erlotinib, Lapatinib, PF.02341066, and Tipifarnib, while showing higher IC50 values for ATRA, Axitinib, and AZD.2281, revealing a significant differential drug sensitivity between the high and low PGRS groups (**Figure 10H**). The study outcomes provided a potential basis for the selection of clinical treatment strategies.

## Discussion

GBM was widely recognized as one of the most challenging malignant tumors due to its high heterogeneity, aggressive invasiveness, high recurrence rate, and treatment difficulty (16). The treatment difficulties stemmed from its complex pathogenesis and multiple biological characteristics, including high heterogeneity, the presence of stem-like cells, along with the generation of an immunosuppressive milieu. These features intertwined and collectively created significant obstacles to treatment. The tumor’s high heterogeneity not only led to poor efficacy of targeted therapies but also caused GBM to develop intrinsic resistance to most existing anticancer drugs (12). At the same time, the stem-like cell population within the TME further exacerbated multidrug resistance (16, 76), while the BBB severely limited the effective delivery of therapeutic drugs (6, 12). Even more troublesome, tumor cells escaped immune surveillance by shaping an immunosuppressive microenvironment (77, 78), making it highly prone to recurrence even after aggressive treatment. These factors worked together, causing the current comprehensive treatment regimen, primarily based on surgical resection combined with radiotherapy and chemotherapy, to only extend survival to a certain extent, but the patient’s prognosis remained very poor. In the face of this dire situation, developing new therapeutic strategies capable of overcoming tumor heterogeneity, breaching the BBB, and regulating the immune microenvironment became an urgent direction of research (79, 80).

Since GBM is fundamentally a type of glioma (16), we specifically focused on the biological characteristics of glioma cells. The findings revealed that glioma cells exhibited significantly higher heterogeneity, proliferative activity, and metabolic properties compared to other cell types, prompting us to conduct a more in-depth analysis of their cellular subtypes. Utilizing scRNA-seq, we identified 9 distinct glioma cell subtypes. Among these, the C2 *PCLAF*+ subtype displayed the most unique proliferative and metabolic features. From a metabolic perspective, the C2 *PCLAF*+ subtype showed significantly markedly enhanced activity in key metabolic pathways such as oxidative phosphorylation, glycolysis/gluconeogenesis, and pyruvate metabolism. This not only revealed its characteristic of maintaining energy supply through the synergy of aerobic glycolysis and mitochondrial metabolism but also directly suggested that this subtype might drive treatment resistance through metabolic reprogramming (81). Specifically, the elevated glycolytic activity helped GBM cells survive in hypoxic microenvironments by rapidly generating ATP and NADH, and provided substrates for DNA repair after chemotherapy. Meanwhile, the enhanced oxidative phosphorylation inhibited apoptotic signals by maintaining mitochondrial membrane potential, thereby resisting cell death induced by radiotherapy or targeted therapy. Existing studies confirmed that the enhancement of glycolysis in GBM was closely related to temozolomide resistance (82). In ST 1 slide, MIA and TransferData analysis demonstrated the unique spatial distribution pattern of C2 *PCLAF*+ subtype in GBM tissue, suggesting that this subtype might establish specific tumor niches to promote recurrence and drug resistance. In ST 2 slide, RCTD analysis showed that high-expression regions of C2 *PCLAF*+ subtype exhibited significant co-localization with spatial expression patterns of *PCLAF* gene, and its distribution characteristics closely matched areas of high heterogeneity and proliferative activity. These observations were consistent with single-cell sequencing data, further supporting the crucial role of C2 *PCLAF*+ subtype in rapid tumor proliferation and invasive growth. Moreover, the spatial distribution of this subtype within tumor tissue was not random but rather preferentially enriched in specific microenvironmental regions, which might be associated with its metabolic adaptability and treatment resistance capabilities.

The C2 *PCLAF*+ subtype played a central regulatory role in the differentiation of GBM, driving tumor heterogeneity evolution through its higher stemness levels and early differentiation potential. This subtype exhibited significant stem cell-like characteristics, marked by the high expression of key stemness genes such as *EZH2*, *LGR5*, *NOTCH1*, and *ABCG2*, which together formed a core regulatory network maintaining tumor stem cell properties. Slingshot analysis further confirmed that all 4 differentiation lineages originated from the C2 *PCLAF*+ subtype, suggesting it might be a key origin subtype driving the multi-directional differentiation of GBM. Furthermore, the functional characteristics of the 4 lineages revealed major biological processes during GBM differentiation. These findings systematically delineated the dynamic evolutionary landscape of GBM, providing new theoretical foundations for understanding the mechanisms underlying tumor heterogeneity formation.

By analyzing intercellular communication patterns, we found that in the MK signaling network, the communication likelihood between the C2 *PCLAF*+ subtype and fibroblasts was significantly increased. The MK signaling pathway exhibited significant functional heterogeneity across different tumor types: In cervical cancer, this pathway synergistically promoted HPV-related progression through ECM remodeling mediated by cancer-associated fibroblasts and immunosuppression (83); in hepatocellular carcinoma, it promoted angiogenesis and metastasis, and coupled lipid metabolism to drive immunosuppression (84); and in lung adenocarcinoma, it collaboratively drove tumor progression with EGFR signaling and induced T cell rejection (85). Previous studies have demonstrated that the MK signaling pathway played a pivotal role in the malignant progression of GBM through multiple mechanisms, including mediating TME remodeling, inducing the formation of an immunosuppressive microenvironment, and driving malignant tumor behaviors (86). Of particular importance, we identified that both the key ligand MDK and its receptor LRP1 in the MK signaling pathway exhibited high expression characteristics in both C2 *PCLAF*+ subtypes and fibroblasts. This pivotal discovery prompted us to focus our investigation on the MDK-LRP1 signaling axis. Ultimately, we demonstrated that the C2 *PCLAF*+ subtype established specific interaction mechanisms with fibroblasts in the TME through the MDK-LRP1 ligand–receptor pair.

To elucidate the transcriptional regulatory mechanisms of the C2 *PCLAF*+ subtype in GBM, we systematically analyzed its specific transcriptional regulatory network. When further dissecting the core regulatory mechanisms of the C2 *PCLAF*+ subtype, we identified the top 5 TFs with the highest specificity scores in this subtype. Notably, the significant upregulation of YEATS4 in the C2 *PCLAF*+ subtype suggested its potential critical role in GBM progression. Current studies demonstrated that YEATS4 (GAS41), as a crucial TF (87, 88), played a pivotal role in the occurrence and development of various tumors. In colorectal cancer, YEATS4 drove tumor cell proliferation by promoting cell cycle transition and inhibiting apoptosis (89); in pancreatic cancer, YEATS4 enhanced the stemness of tumor cells and mediated resistance to gemcitabine by binding to H2A.Z.2 and activating the Notch1 signaling pathway (90); in non-small cell lung cancer, YEATS4 amplification antagonized cellular senescence by inhibiting the p53-p21 pathway, promoted proliferation, and led to cisplatin resistance (91). It was worth noting that YEATS4 also played a crucial role in the malignant progression of GBM. For instance, studies showed that miR-203 could affect the proliferation and migration of GBM cells by regulating the GAS41/miR-10b axis (87). Therefore, it was necessary for us to further explore the specific function of YEATS4 in GBM and verify its specific role in regulating the proliferation and malignant progression of GBM cells through *in vitro* experiments.

The experimental results demonstrated that knockdown of *YEATS4* in two GBM cell lines, LN229 and A1207, led to significant suppression of multiple malignant phenotypes. Specifically, *YEATS4* knockdown resulted in decreased cell viability, markedly reduced colony-forming capacity, impaired cell proliferation, as well as inhibited migratory and invasive abilities in GBM cells. Collectively, these experimental findings indicated that *YEATS4* played an essential contributor in regulating the proliferation, migration and invasion of GBM cells, potentially promoting tumor malignancy through modulation of these key biological processes. Therefore, *YEATS4* might represent a potential therapeutic target for GBM treatment.

To establish a reliable molecular marker for predicting the prognosis of GBM, we formulated a prognostic risk score model founded on C2 *PCLAF*+ glioma cells, which effectively distinguished different patient groups with different prognosis risks. Based on this, we further investigated the immune microenvironment characteristics of GBM, and the differences in the composition of immune cells reflected the heterogeneity of the TME between the high and low PGRS groups. The important point is that a strong positive correlation was observed between the key prognostic genes (*IRF7* and Macrophages M1, and *IKZF3* and B cells naive), suggesting that these genes may regulate the functions of specific immune cells (such as macrophage polarization and B cell activation status) to shape the immunosuppressive or activating phenotype of the TME (92–94). Genomic analysis indicated that the high PGRS group exhibited a more active somatic mutation landscape and more extensive CNVs, which might influence their regulatory functions through dosage effects. This not only drives the malignant progression of tumors but may also indirectly shape the immunosuppressive TME by influencing cytokine secretion or interferon signaling pathways. Evaluation of immunotherapy response further supported the potential sensitivity of the high PGRS group to immunotherapy, reflecting a stronger immune response capacity. Analysis of immune checkpoint genes reinforced these findings, with several immune checkpoint-related genes (e.g., *CD276*, *CD44*, and *C10orf54*) significantly upregulated in the high PGRS group, potentially promoting immune evasion and influencing immunotherapy efficacy.

To comprehensively characterize the molecular differences across PGRS stratification groups, we analyzed DEGs and their functional enrichment profiles. Of particular note was the marked activation of the IL-17 signaling pathway (95, 96), in agreement with the pro-inflammatory microenvironment observed in the high PGRS group. Furthermore, the drug sensitivity analysis provided direct clues for individualized treatment based on the PGRS. In summary, the PGRS scoring system was not only a prognostic tool, but also its underlying immune cell map and the strong correlation between key genes and immune cells profoundly revealed the inherent immunosuppressive characteristics of GBM. This immunosuppressive microenvironment was a key barrier that drove tumor progression and treatment resistance. Our findings emphasized the importance of combined treatment strategies: that is, while directly killing tumor cells, it was necessary to actively intervene in the TME, reverse immunosuppression (such as targeting immunosuppressive cells or their secreted factors), release the suppressed anti-tumor immune response, so as to effectively improve treatment efficacy and overcome drug resistance.

This study thoroughly explored the unique molecular characteristics and spatial distribution patterns of the C2 *PCLAF*+ subtype in GBM and clarified its critical role in tumor progression, metabolic reprogramming, and cell death regulation. More importantly, based on the potential mechanisms of this subtype in GBM heterogeneity and treatment resistance, we further discussed potential clinical translational directions, providing important insights for the development of novel therapeutic strategies. Specifically, we proposed subtype-specific targeted therapies for C2 *PCLAF*+, particularly targeting its metabolic features (such as the development of glycolysis inhibitors) and key regulatory factor YEATS4. Meanwhile, the predictive models constructed by integrating ST data have shown potential clinical application value. They can not only be used to predict patients’ sensitivity to specific treatment regimens but also provide new technical means for real-time monitoring of the dynamic evolution of the TME during treatment. Based on this, we suggested a combined therapeutic strategy to block the MDK-LRP1 signaling axis, aimed at disrupting malignant cell-niche interplay. Finally, we proposed a personalized treatment strategy based on PGRS stratification. For instance, in clinical practice, the model can be integrated into the preoperative assessment and follow-up procedures, and combined with traditional imaging and histopathological information. Additionally, efforts can be made to enhance the coupling development of drug combination strategies and immune microenvironment regulation methods. For example, high PGRS patients can be included in the combined treatment plans of immunostimulants or metabolic intervention drugs to break through the current bottleneck of treatment resistance.

However, this research had certain limitations that merit consideration. Although the study highlighted the critical role of the C2 *PCLAF*+ subtype, other subtypes might also play important roles at different stages of GBM or in different microenvironments. Future research will need to further explore the functional characteristics of other subtypes to furnish a broader perspective on GBM heterogeneity. Additionally, the ST analysis was conducted using only two tissue sections, which limited the universality of the spatial localization results of the C2 *PCLAF*+ subtype. Future studies should expand the spatial dataset. Some of the findings in this study were validated through *in vitro* experiments; however, *in vitro* models might not fully replicate the complex *in vivo* TME, and key rescue experiments (such as overexpression of *YEATS4* after siRNA treatment) are absent to confirm the causal relationship of the *PCLAF*+/*YEATS4* axis. Moreover, the downstream targets of YEATS4 (such as *PCLAF* or cell cycle regulators) still required further validation at the protein level. The tumor immune microenvironment was analyzed using only computational deconvolution methods, and while these methods were feasible, the reliability of the immune feature analysis would be significantly enhanced if they could be verified through experimental means. The complexity of the tumor immune microenvironment was another area that needed further exploration (97–100), particularly the interactions between immune cells, immune evasion mechanisms, and immune tolerance, all of which remained to be investigated.

## Conclusion

This study employed scRNA-seq and ST technologies to systematically analyze the molecular characteristics and spatial heterogeneity distribution patterns of the C2 *PCLAF*+ subtype in GBM. Through integrated multi-omics analysis, we found that the C2 *PCLAF*+ subtype exhibited significantly enhanced proliferative activity, unique metabolic reprogramming features, and aberrant cell death regulation mechanisms, all of which contributed to rapid tumor growth and the development of a drug-resistant phenotype. Notably, *in vitro* functional experiments confirmed the critical role of *YEATS4* in promoting GBM progression, providing a potential new therapeutic target for targeted treatment strategies. Based on the molecular characteristics of the C2 *PCLAF*+ subtype, we successfully constructed an effective prognostic risk score model and integrated genomic, immune microenvironment, and drug sensitivity data. These findings offered important theoretical insights and practical guidance for a fuller comprehension of the molecular pathogenesis of GBM and the development of personalized treatment strategies.

## Data Availability

The original contributions presented in the study are included in the article/**Supplementary Material**. Further inquiries can be directed to the corresponding author/s.

## Supplementary material

Supplementary Figure 1 Spatial transcriptome atlas. **(A, B)** ST feature maps showed the spatial expression pattern of the C2 *PCLAF*+ subtype and *PCLAF* gene on ST 2 slide. **(C)** ST feature maps revealed spatial distributions of nCount-Spatial, nFeature-Spatial, G2/M.Score, and S.Score across ST 2 slide.
