# Supplementary figures and images for: Single-cell and spatial atlas of glioblastoma heterogeneity: characterizing the PCLAF+ subtype and YEATS4’s oncogenic role
Source: Front Immunol. 2025 Jul 25;16:1614549. doi: 10.3389/fimmu.2025.1614549 (PMC12331606; doi:10.3389/fimmu.2025.1614549)

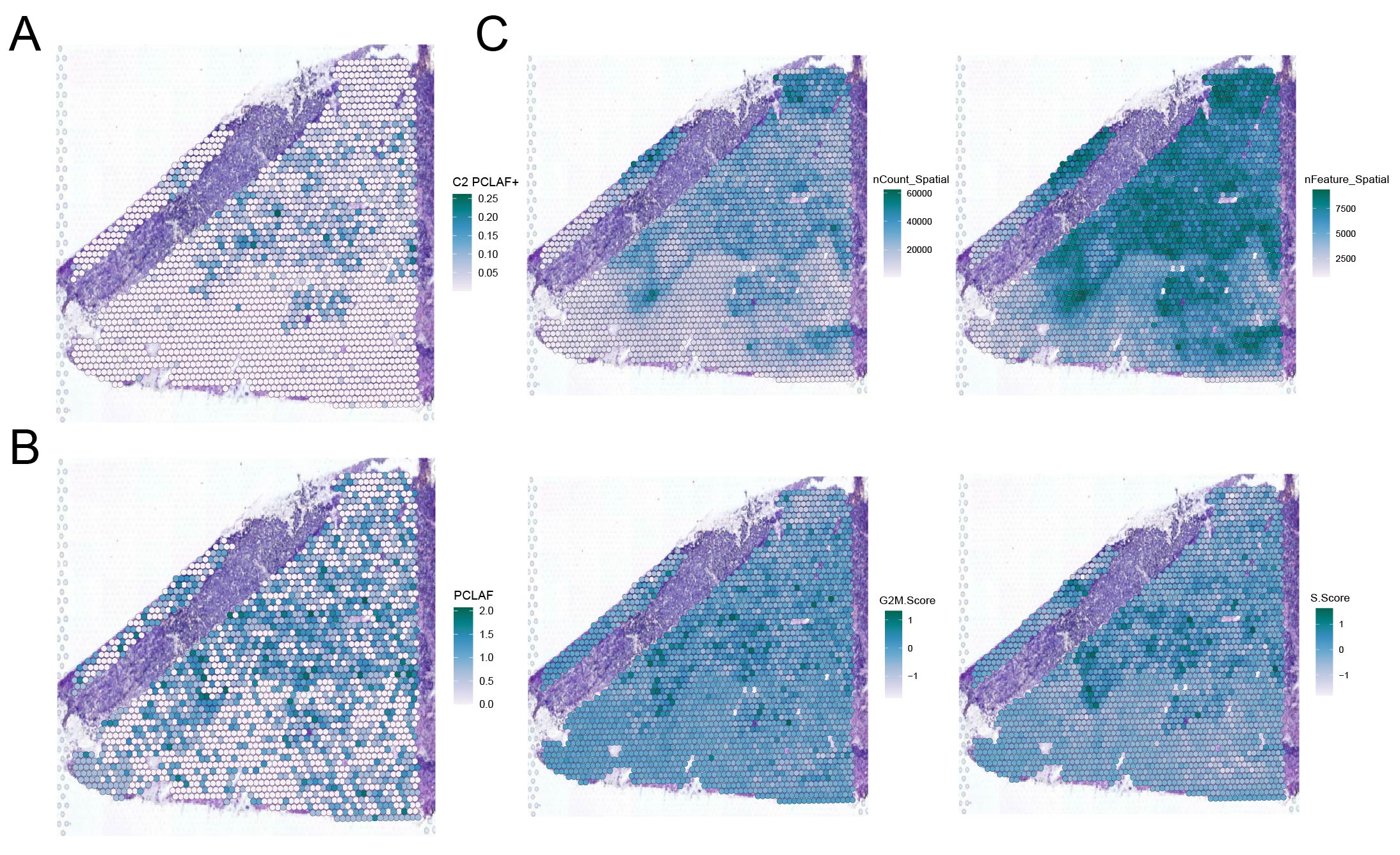

Supplement: Supplementary Figure 1 — Spatial transcriptome atlas. (A, B) ST feature maps showed the spatial expression pattern of the C2 PCLAF+ subtype and PCLAF gene on ST 2 slide. (C) ST feature maps revealed spatial distributions of nCount-Spatial, nFeature-Spatial, G2/M.Score, and S.Score across ST 2 slide. [file Image1.jpeg]
